# Effect of Harm Anchors in Visual Displays of Test Results on Patient Perceptions of Urgency About Near-Normal Values: Experimental Study

**DOI:** 10.2196/jmir.8889

**Published:** 2018-03-26

**Authors:** Brian J Zikmund-Fisher, Aaron M Scherer, Holly O Witteman, Jacob B Solomon, Nicole L Exe, Angela Fagerlin

**Affiliations:** ^1^ Department of Health Behavior and Health Education University of Michigan Ann Arbor, MI United States; ^2^ Department of Internal Medicine University of Michigan Ann Arbor, MI United States; ^3^ Center for Bioethics and Social Sciences in Medicine University of Michigan Ann Arbor, MI United States; ^4^ Department of Internal Medicine University of Iowa Iowa City, IA United States; ^5^ Department of Family and Emergency Medicine Laval University Quebec City, QC Canada; ^6^ Office of Education and Professional Development Faculty of Medicine Laval University Quebec City, QC Canada; ^7^ Population Health and Optimal Health Practices Research Unit Research Center of the CHU de Québec-Université Laval Quebec City, QC Canada; ^8^ Laval University Research Institute for Primary Care and Health Services Quebec City, QC Canada; ^9^ Department of Population Health Sciences University of Utah Salt Lake City, UT United States; ^10^ Salt Lake City Veterans Affairs Center for Informatics Decision Enhancement and Surveillance Salt Lake City, UT United States

**Keywords:** decision making, education of patients, electronic health record, computer graphics, clinical laboratory information systems

## Abstract

**Background:**

Patient-facing displays of laboratory test results typically provide patients with one reference point (the “standard range”).

**Objective:**

To test the effect of including an additional harm anchor reference point in visual displays of laboratory test results, which indicates how far outside of the standard range values would need to be in order to suggest substantial patient risk.

**Methods:**

Using a demographically diverse, online sample, we compared the reactions of 1618 adults in the United States who viewed visual line displays that included both standard range and harm anchor reference points (“Many doctors are not concerned until here”) to displays that included either (1) only a standard range, (2) standard range plus evaluative categories (eg, “borderline high”), or (3) a color gradient showing degree of deviation from the standard range.

**Results:**

Providing the harm anchor reference point significantly reduced perceived urgency of close-to-normal alanine aminotransferase and creatinine results (*P* values <.001) but not generally for platelet count results. Notably, display type did not significantly alter perceptions of more extreme results in potentially harmful ranges. Harm anchors also substantially reduced the number of participants who wanted to contact their doctor urgently or go to the hospital about these test results.

**Conclusions:**

Presenting patients with evaluative cues regarding when test results become clinically concerning can reduce the perceived urgency of out-of-range results that do not require immediate clinical action.

## Introduction

Patients can increasingly view their laboratory test results directly via patient portals of electronic health record (EHR) systems [1]. While patients value such information to enable self-management and support informed patient-provider interactions [2-5], access to test results does not guarantee that patients can understand or use that information to improve their health or their care [6]. In particular, most current EHR patient portals present test results to patients in tables [7], which is a format that is difficult for many patients to interpret, especially those with lower numeracy or literacy skills [8]. In addition, EHR portals typically only provide patients with one reference point (the standard range) to aid in interpreting such data. Patients who receive out-of-range test values may have little idea how alarmed they should, or should not, feel [5,6,9,10]. Consequently, patients may call their doctor for an urgent appointment or even go to the hospital for something that would be more appropriately managed through regular follow-up visits [10].

In order to improve patient understanding of laboratory test results, our research team used user-centered design principles to develop several visual number line formats for presenting these types of data [11]. In an experimental test, displaying test results in these number line formats instead of tables increased user sensitivity to test result variations [12]. We also showed that many people interpret all results outside of the standard range as equally urgent, even though many slightly out-of-range results are not, in fact, clinically concerning.

The core problem that patients face is one of information evaluability [13,14]. Many patients lack training and experience with most laboratory tests, so they cannot necessarily map a particular test result to its meaning (ie, how good or bad it is, or how much risk it represents). As long as most patients lack meaningful reference points beyond the standard range, they will struggle to discriminate between different types of non-normal test results.

Here, we present results from additional data collected at the same time as the previous study in which we tested a visual display format that added a second reference point to indicate how far outside of the standard range a test value needs to be to become clinically concerning. Our objective was to determine whether providing such “harm anchors” would reduce the perceived urgency of near-normal values without significantly altering perceptions of extreme values, thereby increasing overall sensitivity to test result variations.

## Methods

Participants were a demographically diverse, stratified random sample of adults in the United States recruited during May 2016 from a panel of Internet users, and the survey was administered by Survey Sampling International. The design, sampling process, data management procedures, and outcome measures received exempt status approval from the University of Michigan Health Sciences and Behavioral Sciences Institutional Review Board.

Participants were asked to imagine that they were using an online EHR portal to view laboratory test results and then saw three specific test results (platelet count, alanine aminotransferase [ALT], and serum creatinine). Each test result was initially shown as slightly outside of the standard range and then as a more extreme test result. All participants viewed a platelet count of 135 x 10^9^/L and then 25 x 10^9^/L, an ALT value of 80 U/L and then 360 U/L, and a creatinine value of 2.2 mg/dL and then 3.4 mg/dL.

**Figure 1:**
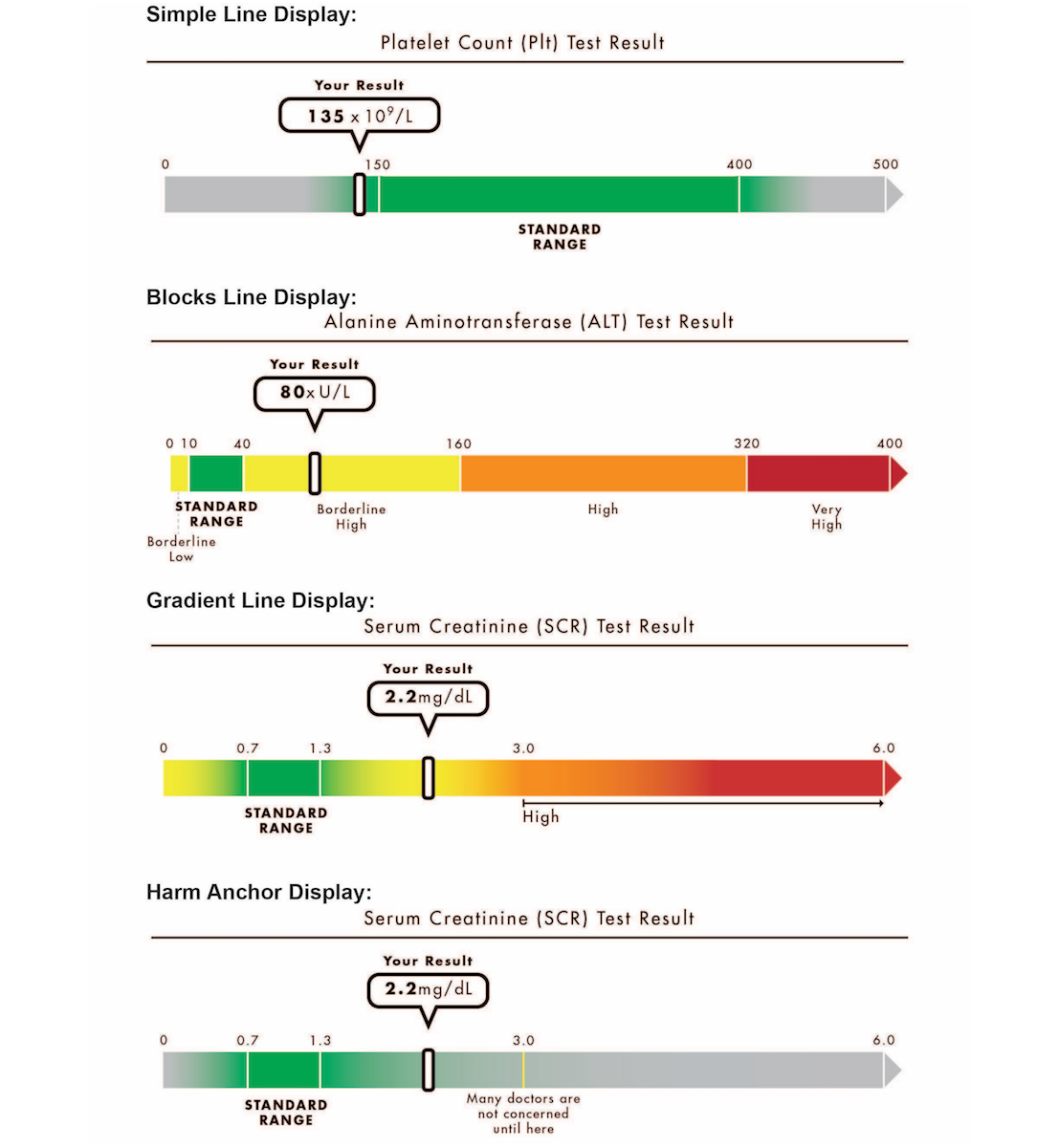
Examples of each of the four visual display formats and three tests included in this study.

In a previous paper, we compared participant reactions to three visual number line graph displays (Figure 1): simple line displays that show the standard range and endpoints, but have no other visual reference points; blocks line displays that included color-coded evaluative categories (eg, “borderline high”); and gradient line displays that used a color gradient to indicate the extent of deviation from the standard range [12]. The range of values shown and the evaluative labels and categories displayed were selected as plausible values based on input from several clinician members of our research team.

In this analysis, we compared the results from those three previously published conditions to data collected at the same time from an additional (randomized) group of participants. These participants received displays that included an added harm anchor (ie, a threshold line outside of the standard range labeled, “many doctors are not concerned until here”) but were otherwise identical to the simple line displays (see Figure 1, bottom image). This language was developed through several iterations of pretesting with patients. Based on consultations with multiple clinician collaborators, we selected the anchor levels shown (platelets=100 x 10^9^/L; ALT=160 U/L; creatinine=3.0 mg/dL) as plausible approximations of the point at which nonnormal values require more urgent attention for most patients.

The primary outcome measure was respondents’ subjective sense of urgency to the displayed test results. We averaged respondents’ responses to two questions: “How alarming does this result feel to you?” and, “How urgent of an issue is this result?” (both measured on 6-point Likert scales; 1=*not at all*, 6=*very*). The resulting measure of subjective urgency showed high reliability for all tests and test results (Cronbach alpha=.91 to .95). We measured behavioral intentions by asking whether respondents would initiate a new contact with a health professional (eg, by calling their doctor for an urgent appointment or going to a hospital) versus either waiting until their next regular appointment or doing nothing (for question details, see Zikmund-Fisher, et al [12]). To measure display format preferences, we used respondents’ average responses on a set of four questions (Cronbach alpha=.87) that asked how well the images described the results, how helpful they were, whether respondents would trust the images, and whether respondents would like to see results presented in these formats (all measured using 5-point Likert scales) [12]. We report one-way analyses of variance (ANOVAs) with Bonferroni corrections to compare ratings of perceived urgency and user preferences, and chi-squared tests to compare willingness to wait. All analyses were performed using STATA 14 [15]. All tests of significance were two-sided and used alpha=.05.

## Results

A total of 1618 adult participants (aged 19 to 89 years) completed the survey and were randomized to the experimental conditions examined here. See Table 1 for respondent characteristics.

As shown in Table 2, providing the harm anchor reference point on the visual display significantly reduced perceived urgency of the close-to-normal ALT and creatinine results compared to all three other display formats (all *P* values <.001) without significantly altering perceptions of more extreme results in potentially harmful ranges. For platelet count results, however, we observed only a mildly significant difference between the harm anchor display and the blocks display. Use of the harm anchor labels also substantially reduced the number of participants who wanted to contact their doctor urgently or go to the hospital when shown near-normal ALT or creatinine test results (Table 3).

Overall, there were no significant differences in participants’ preferences among the four display types (Harm Anchor mean=3.77 vs Simple Line mean=3.62, Blocks Line mean=3.76, Gradient Line mean=3.68; ANOVA F(3, 1615)=2.33, *P*=.07).

**Table 1:** Respondent characteristics.

Characteristic		Value
Age, mean (range)		48.8 (19,89)
**Gender, n (%)**		
	Male	769 (47.6)
	Female	842 (52.1)
	Transgender	4 (0.25)
**Race, n (%)**		
	White	1255 (77.8)
	Black	213 (13.2)
	Asian	64 (4.0)
	Native American	13 (0.8)
	Other / Multi-race	69 (4.3)
	Hispanic	212 (13.2)
Bachelor’s degree or higher education, n (%)		810 (50.1)
**Current health, n (%)**		
	Excellent	248 (15.3)
	Very good	605 (37.4)
	Good	522 (32.3)
	Fair	213 (13.2)
	Poor	29 (1.8)

**Table 2:** Perceived urgency of near-normal and more extreme test results, by display type. Perceived urgency was measured on a 1-6 scale, with higher numbers corresponding to greater perceived urgency. *P* values were calculated by post-hoc comparisons following one-way analyses of variance with Bonferroni corrections for multiple comparisons. ALT: alanine aminotransferase.

Test result		Harm Anchor	Simple Line		Blocks Line		Gradient Line	
			Rating	*P* value^a^	Rating	*P* value^a^	Rating	*P* value^a^
**Near-normal results**								
	Platelets=135 x 10^9^/L	3.66	3.72	>.99	3.94	.016	3.73	>.99
	ALT=80 U/L	3.08	4.00	<.001	3.96	<.001	3.56	<.001
	Creatinine=2.2 mg/dL	3.52	4.09	<.001	3.99	<.001	3.91	<.001
**Extreme results**								
	Platelets=25 x 10^9^/L	5.09	5.26	.32	5.20	>.99	5.30	.10
	ALT=360 U/L	5.32	5.44	.78	5.35	>.99	5.39	.10
	Creatinine=3.4 mg/dL	4.71	4.81	>.99	4.58	.48	4.74	>.99

^a^*P* values reported are for comparisons to the harm anchor condition.

**Table 3:** Percentage of participants reporting intentions to contact their doctor urgently or go to the hospital based on their near-normal test results, by display type. ALT: alanine aminotransferase.

Test result	Harm Anchor	Simple Line	Blocks Line	Gradient Line	Overall test	
					Chi square statistic	*P* value
Platelets=135 x 10^9^/L	44.2%	50.0%	53.3%	51.6%	χ^2^(3)=7.61	.06
ALT=80 U/L	34.7%	55.8%	58.1%	48.2%	χ^2^(3)=53.83	<.001
Creatinine=2.2 mg/dL	35.2%	56.7%	53.5%	52.3%	χ^2^(3)=45.15	<.001

## Discussion

As hypothesized, presenting patients with cues regarding the values at which particular test results become clinically concerning reduced respondents’ perceptions of urgency about certain types of out-of-range results that were not of immediate clinical concern. Our results suggest that including harm anchors in test result communications could, in certain circumstances, provide important benefits to patients by increasing the evaluability of variations among out-of-range results.

As in our previous comparison of visual line displays versus tabular displays [12], the effect size we observed varied substantially across the three tests presented. A possible explanation for this finding is that it is a function of the relative size of the standard reference range, as compared to the range of values shown. The largest effect of harm anchors was observed for ALT tests, which have a narrow reference range within a large range of possible values. By contrast, we observed minimal to no effect of harm anchors on displays of platelet counts; a test for which deviations of 50 to 100 (x 10^9^/L) outside of the standard range (only 20%-40% of the width of the standard range) represent significant changes in patient risk. Thus, harm anchors may be most useful when communicating with patients about unfamiliar tests that can have wide ranges of potential variation.

Operationalization of this idea, however, will require overcoming several challenges. First, harm anchors, by definition, represent clinical judgment, and different clinicians may reasonably disagree regarding the point at which harm threshold should be set [16]. Second, even if harm thresholds could be agreed upon, the point at which a patient (or clinician) should view a test result as requiring urgent action should logically vary based on patient characteristics or medical context (eg, initial diagnosis vs long term management). We also acknowledge the primary limitations of this study: the use of a hypothetical scenario and the testing of the harm anchor concept within the constraints of a particular visual display design. Inclusion of harm anchor information within other types of visual displays or tables might result in different findings than those observed here.

Nonetheless, our results demonstrate that designing displays to inform patients regarding what is dangerous, as opposed to what is considered usual or normal, might offer practical benefits. We suggest that harm anchors or other risk-related reference points should be considered when designing patient-facing displays of health data in order to increase the interpretability of such communications. These types of displays should be most useful in situations where relatively unfamiliar laboratory tests are being conducted for monitoring purposes (eg, monitoring liver or kidney function while on extended medication regimens). These situations are likely to result in mild deviations in test result values that, while important to monitor, are not immediately concerning to clinicians. Enabling patients to know that these mild deviations are not urgent will reduce patient worry and might also minimize unnecessary patient requests for urgent appointments when routine follow-up would be sufficient.

## Abbreviations

ALT: alanine aminotransferase
ANOVA: analysis of variance
EHR: electronic health record
