# Correction: Effect of Harm Anchors in Visual Displays of Test Results on Patient Perceptions of Urgency About Near-Normal Values: Experimental Study

**DOI:** 10.2196/74908

**Published:** 2025-04-02

**Authors:** Brian J Zikmund-Fisher, Aaron M Scherer, Holly O Witteman, Jacob B Solomon, Nicole L Exe, Angela Fagerlin

**Affiliations:** 1 Department of Health Behavior and Health Education University of Michigan Ann Arbor, MI United States; 2 Department of Internal Medicine University of Michigan Ann Arbor, MI United States; 3 Center for Bioethics and Social Sciences in Medicine University of Michigan Ann Arbor, MI United States; 4 Department of Internal Medicine University of Iowa Iowa City, IA United States; 5 Department of Family and Emergency Medicine Laval University Quebec City, QC Canada; 6 Office of Education and Professional Development Faculty of Medicine Laval University Quebec City, QC Canada; 7 Population Health and Optimal Health Practices Research Unit Research Center of the CHU de Québec-Université Laval Quebec City, QC Canada; 8 Laval University Research Institute for Primary Care and Health Services Quebec City, QC Canada; 9 Department of Population Health Sciences University of Utah Salt Lake City, UT United States; 10 Salt Lake City Veterans Affairs Center for Informatics Decision Enhancement and Surveillance Salt Lake City, UT United States

In “Effect of Harm Anchors in Visual Displays of Test Results on Patient Perceptions of Urgency About Near-Normal Values: Experimental Study” (J Med Internet Res 2018;20(3):e98) the authors noted one error.

In Table 2, the row labels for the 3 rows under “Extreme results” have been revised. The row headers that previously stated:

Platelets=135 x 10^9^/L

ALT=80 U/L

Creatinine=2.2 mg/dL

Have been revised to read:

Platelets=25 x 10^9^/L

ALT=360 U/L

Creatinine=3.4 mg/dL

The correction will appear in the online version of the paper on the JMIR Publications website, together with the publication of this correction notice. Because this was made after submission to PubMed, PubMed Central, and other full-text repositories, the corrected article has also been resubmitted to those repositories.

